# A transformer-based generative adversarial network for brain tumor segmentation

**DOI:** 10.3389/fnins.2022.1054948

**Published:** 2022-11-30

**Authors:** Liqun Huang, Enjun Zhu, Long Chen, Zhaoyang Wang, Senchun Chai, Baihai Zhang

**Affiliations:** ^1^The School of Automation, Beijing Institute of Technology, Beijing, China; ^2^Department of Cardiac Surgery, Beijing Anzhen Hospital, Capital Medical University, Beijing, China

**Keywords:** generative adversarial network, transformer, deep learning, automatic segmentation, brain tumor

## Abstract

Brain tumor segmentation remains a challenge in medical image segmentation tasks. With the application of transformer in various computer vision tasks, transformer blocks show the capability of learning long-distance dependency in global space, which is complementary to CNNs. In this paper, we proposed a novel transformer-based generative adversarial network to automatically segment brain tumors with multi-modalities MRI. Our architecture consists of a generator and a discriminator, which is trained in min–max game progress. The generator is based on a typical “U-shaped” encoder–decoder architecture, whose bottom layer is composed of transformer blocks with Resnet. Besides, the generator is trained with deep supervision technology. The discriminator we designed is a CNN-based network with multi-scale *L*_1_ loss, which is proved to be effective for medical semantic image segmentation. To validate the effectiveness of our method, we conducted exclusive experiments on BRATS2015 dataset, achieving comparable or better performance than previous state-of-the-art methods. On additional datasets, including BRATS2018 and BRATS2020, experimental results prove that our technique is capable of generalizing successfully.

## 1. Introduction

Semantic medical image segmentation is an indispensable step in computer-aided diagnosis (Stoitsis et al., 2006; Le, 2017; Razmjooy et al., 2020; Khan et al., 2021). In clinical practice, tumor delineation is usually performed manually or semi-manually, which is time-consuming and labor-intensive. As a result, it is of vital importance to explore automatic volumetric segmentation methods with the help of medical images to accelerate the computer-aided diagnosis. In this paper, we focus on the segmentation of brain tumors with the help of magnetic resonance imaging (MRI) consisting of multi-modality scans. The automatic segmentation of gliomas remains one of the most challenging medical segmentation problems stemming from some aspects, such as arbitrary shape and location, poorly contrasted, and blurred boundary with surrounding issues.

Since the advent of deep learning, Convolutional Neural Networks (CNN) have achieved great success in various computer vision tasks, ranging from classification (LeCun et al., 1998; Krizhevsky et al., 2012; Simonyan and Zisserman, 2014; Szegedy et al., 2015; Huang et al., 2017), object detection (Girshick et al., 2014; Girshick, 2015; Ren et al., 2015; Liu et al., 2016; Redmon et al., 2016; He et al., 2017; Redmon and Farhadi, 2017, 2018; Bochkovskiy et al., 2020) to segmentation (Chen et al., 2014, 2017; Long et al., 2015; Ronneberger et al., 2015; Lin et al., 2017). Fully Convolution Networks (FCN Long et al., 2015) and in particular “U-shaped” encoder–decoder architectures have realized state-of-the-art results in medical semantic segmentation tasks. U-Net (Ronneberger et al., 2015), which consists of symmetric encoder and decoder, uses the skip connections to merge the extracted features from encoder with those from decoder at different resolutions, aiming at recovering the lost details during downsampling. Owing to the impressive results in plenty of medical applications, U-Net and its variants have become the mainstream architectures in medical semantic segmentation.

In spite of their prevalence, FCN-based approaches are incapable of modeling long-range dependency because of its intrinsic limited receptive field and the locality of convolution operations. Inspired by the great success of transformer-based models in Natural Language Processing (NLP) (Devlin et al., 2018; Radford et al., 2018; Liu et al., 2019; Yang et al., 2019; Clark et al., 2020), a growing number of researchers propose to apply the self-attention mechanism to medical image segmentation, attempting to overcome the limitations brought by the inductive bias of convolution, so as to extract the long-range dependency and context–dependent features. Especially, unlike prior convolution operations, transformers encode a sequence of patches and leverage the power of self-attention modules to pre-train on a large-scale dataset for downstream tasks, like Vision Transformer (ViT) (Dosovitskiy et al., 2020) and its variants.

Simultaneously, for the Transformers applied in medical image segmentation, Generative Adversarial Networks (GAN) has revealed great performance in semantic segmentation. In a typical GAN architecture used for segmentation, GAN consists of two competing networks, a discriminator and a generator. The generator learns the capability of contexture representations, minimizing the distance between prediction and masks, while the discriminator on the contrary maximizes the distance to distinguish the difference between them. The two networks are trained in an alternating fashion to improve the performance of the other. Furthermore, some GAN-based methods like SegAN (Xue et al., 2018) achieve more effective segmentation performance than FCN-based approaches.

In this paper, we explore the integrated performance of transformer and generative adversarial network in segmentation tasks and propose a novel transformer-based generative adversarial network for brain tumor segmentation. Owing to the attention mechanism, transformer has a global receptive field from the very first layer to the last layer, instead of focusing solely on the local information from convolution kernel in each layer, thus contributing to the pixel-level classification and being more suitable for medical segmentation tasks. Besides, CNN learns representative features at different resolutions through cascading relationships, while the attention mechanism pays more attention to the relationship between features, thus transformer-based methods are easily-generalized and not completely dependent on the data itself, such as experiments with incomplete images input in Naseer et al. (2021). Inspired by some attempts (Wang W. et al., 2021; Hatamizadeh et al., 2022) of fusing transformer with 3D CNNs, we design an encoder–decoder generator with deep supervision, where both encoder and decoder are 3D CNNs but the bridge of them is composed of transformer blocks with Resnet. In the contrast of typical “U-shaped” decoder–encoder network, our transformer block is designed to replace the traditional convolution-based bottleneck, for the reason that the self-attention mechanism inside transformer can learn long-range contextual representations while the finite kernel size limits the CNN's capability of learning global information. For pixel-wise brain tumor segmentation task, replacing CNN with transformer blocks on the bottleneck contributes to capturing more features from encoder. Inspired by SegAN (Xue et al., 2018), we adopt the multi-scale *L*_1_ loss to our method with only one generator and one discriminator, measuring the distance of the hierarchical features between generated segmentation and ground truth. Experimental results on BRATS2015 dataset show that our method achieves comparable or better performance than some previous state-of-the-art methods. Compared to existing methods, the main contributions of our approach are listed as follows:

- A novel transformer-based generative adversarial network is proposed to address the brain tumor segmentation task with multi-modalities MRI. To enhance the efficiency of brain tumor segmentation, our method incorporates the concepts of “Transformer” and “Generative adversarial”. The generator makes use of the transformer blocks to facilitate the process of learning global contextual representations. As far as we are aware, our work is among the very first ones to explore the combination of transformer and generative adversarial networks and achieve excellent performance in the brain tumor segmentation task.
- Our generator exploits transformer with Resnet module in 3D CNN for segmenting multi-modalities MRI brain tumors. Building upon the encoder–decoder structure, both encoder and decoder in our proposed generator are mainly composed of traditional 3D convolution layers, while the bottom layer of the “U-shaped” structure is a transformer with Resnet module. With Resnet, the transformer block captures both global and local spatial dependencies effectively, thus preparing embedded features for progressive upsampling to full resolution predicted maps.
- Our loss functions are suitable and effectively applied in generator and discriminator. Adopting the idea of deep supervision (Zhu Q. et al., 2017), we take the output of the last three decoder layers of generator to calculate weighted loss for better gradient propagation. Besides, we leverage a CNN-based discriminator to compute multi-scale *L*_1_ norm distance of hierarchical features extracted from ground truth and segmentation maps, respectively.
- The exclusive experimental results evaluated on BRATS2015 dataset show the effectiveness of each part of our proposed methods, including transformer with Resnet module and loss functions. Comparing to existing methods, the proposed method can obtain significant improvements in brain tumor segmentation. Moreover, our method successfully generalizes in other brain tumor segmentation datasets: BRATS2018 and BRATS2020.

The following outlines the structure of this paper: Section 2 reviews the related work. Section 3 presents the detail of our proposed architecture. Section 4 describes the experimental setup and evaluates the performance of our method. Section 5 summarizes this work.

## 2. Related works

### 2.1. Vision transformer

The Transformers were first proposed by Vaswani et al. (2017) on machine translation tasks and achieved a quantity of state-of-the-art results in NLP tasks (Devlin et al., 2018; Radford et al., 2018). Dosovitskiy et al. (2020) then applied Transformers to image classification tasks by directly training a pure Transformer on sequences of image patches as words in NLP, and achieved state-of-the-art benchmarks on the ImageNet dataset. In object detection, Carion et al. (2020) proposed transformer-based DETR, a transformer encoder–decoder architecture, which demonstrated accuracy and run-time performance on par with the highly optimized Faster R-CNN (Ren et al., 2015) on COCO dataset.

Recently, various approaches were proposed to explore the applications of the transformer-based model for semantic segmentation tasks. Chen et al. (2021) proposed TransUNet, which added transformer layers to the encoder to achieve competitive performance for 2D multi-organ medical image segmentation. As for 3D medical image segmentation, Wang W. et al. (2021) exploited Transformer in 3D CNN for segmenting MRI brain tumors and proposed to use a transformer in the bottleneck of “U-shaped” network on BRATS2019 and BRATS2020 datasets. Similarly, Hatamizadeh et al. (2022) proposed an encoder–decoder network named UNETR, which employed transformer modules as the encoder and CNN modules as the decoder, for the brain tumor and spleen volumetric medical image segmentation.

Compared to these approaches above, our method is tailored for 3D segmentation and is based on generative adversarial network. Our generator produces sequences fed into transformer by utilizing a backbone encoder–decoder CNN, where the transformer with Resnet module is placed in the bottleneck. With Resnet, the encoder captures features not only from CNN-based encoder but also from transformer blocks. Moreover, the last three output layers of the encoder are considered to calculate the loss function for better performance. Networks like UNETR employ transformer layers as encoder in low-dimension semantic level, and taking this network as backbone in our method without pre-training easily leads to model collapse during the adversarial training phase. Therefore, we do not choose these networks as our backbone. We find that taking transformer as encoder in low-dimension semantic level needs quantities of pre-training tasks on other datasets to get good results, like TransUNet and UNETR above. As shown in our experiments Section 4.6, transformer-based encoder in low-dimension semantic level performances inferior to CNN-based one when training from scratch. Therefore, we choose to apply transformer only in bottleneck, and remain the low-dimension encode layers as convolutional layers. In this way, we can train from scratch, meanwhile achieving good performance.

### 2.2. Generative adversarial networks

The GAN (Goodfellow et al., 2014) is originally introduced for image generation (Mirza and Osindero, 2014; Chen et al., 2016; Odena et al., 2017; Zhu J.-Y. et al., 2017), making the core idea of competing training with a generator and a discriminator, respectively, known outside of fixed circle. However, there exists a problem that it is troublesome for the original GAN to remain in a stable state, hence making us cautious to balance the training level of the generator and the discriminator in practice. Arjovsky et al. proposed Wasserstein GAN (WGAN) as a thorough solution to the instability by replacing the Kullback-Leibler (KL) divergence with the Earth Mover (EM) distance.

Various methods (Isola et al., 2017; Han et al., 2018; Xue et al., 2018; Choi et al., 2019; Dong et al., 2019; Oh et al., 2020; Ding et al., 2021; He et al., 2021; Nishio et al., 2021; Wang T. et al., 2021; Zhan et al., 2021; Asis-Cruz et al., 2022) were proposed to explore the possibility of GAN in medical image segmentation. Xue et al. (2018) used U-Net as the generator and proposed a multi-scale *L*_1_ loss to minimize the distance of the feature maps of predictions and masks for the medical image segmentation of brain tumors. Oh et al. (2020) took residual blocks into account under the framework of pix2pix (Isola et al., 2017) and segmented the white matter in FDG-PET images. Ding et al. (2021) took an encoder–decoder network as the generator and designed a discriminator based on Condition GAN (CGAN) on BRATS2015 dataset, adopting the image labels as the extra input.

Unlike these approaches, our method incorporates the concepts of “Transformer” and “GAN.” Our discriminator is based on CNN instead of transformer. In our opinion, owing to the attention mechanism inside transformer, transformer has a more global receptive field than CNN with limited kernel size, thus contributing to pixel-level classification and being more suitable for medical segmentation tasks. However, for image-level medical classification, transformer-based discriminator seems to be less appropriate for its weakness of requiring huge datasets to support pre-training, while CNN is strong enough for classification tasks without pre-training. Motivated by viewpoints above, in our method, the transformer-based generator and CNN-based discriminator are combined to facilitate the progress of segmentation under the supervision of a multi-scale *L*_1_ loss.

## 3. Materials and methods

### 3.1. Overall architecture

**Figure 1:**
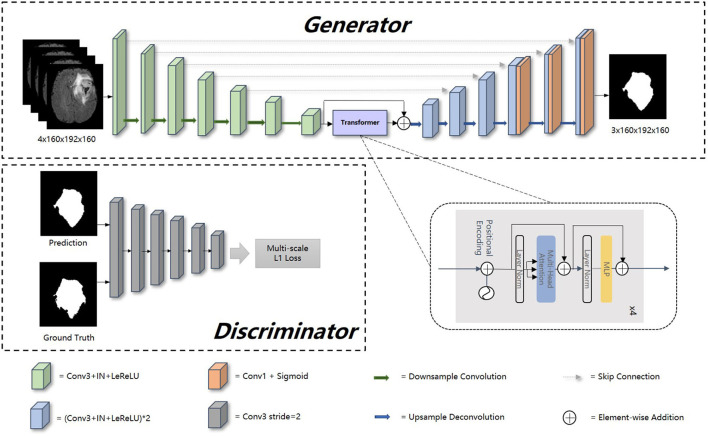
Overall architecture of our proposed method. In this figure, “Conv1” represents convolutional layer with kernel 1 × 1 × 1, “Conv3” with kernel 3 × 3 × 3, “IN” represents InstanceNorm layer, “LeReLU” means LeakyReLU activation layer.

The overview of our proposed model is presented in Figure 1. Our framework consists of a generator and discriminator for competing training. The generator G is a transformer-based encoder–decoder architecture. Given a multi modalities (T1, T1c, T2, and FLAIR) MRI scan *X* ∈ *R*^*C* × *H* × *W* × *D*^ with 3D resolution (H, W, D) and C channels, we utilize 3D CNN-based down-sampling encoder to produce high dimension semantic feature maps, and then these semantic information flow to 3D CNN-based up-sampling decoder through the intermediate Transformer block with Resnet (He et al., 2016). With skip connection, the long-range and short-range spatial relations extracted by encoder from each stage flow to the decoder. For deep supervision (Zhu Q. et al., 2017), the output of decoder consists of three parts: the output of last three convolution layers after sigmoid. Inspired by Xue et al. (2018), the discriminator D we used has the similar structure as encoder in G, extracting hierarchical feature maps from ground truth (GT) and prediction separately to compute multi-scale *L*_1_ loss.

### 3.2. Generator

Encoder is the contracting path which has seven spatial levels. Patches of size 160 × 192 × 160 with four channels are randomly cropped from brain tumor images as input, followed by six down-sampling layers with 3D 3 × 3 × 3 convolution (stride = 2). Each convolution operation is followed by an Instance Normalization (IN) layer and a LeakyReLU activation layer.

At the bottom of the encoder, we leverage the transformer with Resnet module to model the long-distance dependency in a global space. The feature maps produced by the encoder is sequenced first and then create the feature embeddings by simply fusing the learnable position embeddings with sequenced feature map by element-wise addition. After the position embeddings, we introduce L transformer layers to extract the long-range dependency and context dependent features. Each transformer layer consists of a Multi-Head Attention (MHA) block after layer normalization (LN) and a feed forward network (FFN) after layer normalization. In attention block, the input sequence is fed into three convolution layers to produce three metrics: queries Q, keys K and values V. To combine the advantages of both CNN and Transformer, we simply short cut the input and output of Transformer block. Thus, as in Vaswani et al. (2017) and Wang W. et al. (2021), given the input X, the output of the transformer with Resnet module Y can be calculated by:

(1) $$\begin{array}{l} Y=x+y_{L} \end{array}$$

(2) $$\begin{array}{l} y_{i}=FFN\left(LN\left(y_{i}^{{\;}^{\prime }}\right)\right)+y_{i}^{{\;}^{\prime }} \end{array}$$

(3) $$\begin{array}{l} y_{i}^{{\;}^{\prime }}=MHA\left(LN\left(y_{i-1}\right)\right)+y_{i-1} \end{array}$$

(4) $$\begin{array}{l} MHA\left(Q,K,V\right)=Concat\left(head_{1},\ldots ,head_{h}\right)W^{O} \end{array}$$

(5) $$\begin{array}{l} head_{i}=Attention\left(Q,K,V\right)=softmax\left(QK^{T}/{\sqrt{d}}_{k}\right)V \end{array}$$

where *y*_*i*_ denotes the output of *i*th (*i* ∈ [1, 2, …, *L*]) Transformer layer, *y*_0_ denotes *X*, *W*^*O*^ are projection metrics, *d*_*k*_ denotes the dimension of *K*.

Unlike the encoder, the decoder uses 3D 2 × 2 × 2 transpose convolution for up-sampling, followed by skip connection and two 3D 3 × 3 × 3 convolution layers. For a better gradient flow and a better supervision performance, a technology called deep supervision is introduced to utilize the last three decoder levels to calculate loss function. Concretely, we downsampled the GT to the same resolution with these outputs, thus making weighted sum of loss functions in different levels.

The detailed structure of our transformer-based generator is presented in Table 1. In the encoder part, patches of size 160 × 192 × 160 voxels with four channels are randomly cropped from the original brain tumor images as input. At each level, there are two successive 3 × 3 × 3 unbiased convolution layers followed by normalization, activation layers and dropout layers. Beginning from the second level, the resolution of the feature maps is reduced by a factor of 2. These features, e.g., areas of white matter, edges of brain, dots and lines, etc., are extracted by sufficient convolution kernels for next blocks. The transformer block enriches the global contextual representation based on the attention mechanism, forcing features located in the desired regions unchanged while suppressing those in other regions. The shortcut branch crossing the transformer block fusing the features from both encoder part and transformer block by element-wise addition, indicating that our generator is capable of learning short-range and long-range spatial relations with neither extra parameter nor computation complexity. According to the attributes of Resnet (He et al., 2016), *y* = *f*(*x*) + *x*, where *f*(*x*) in our method represents transformer blocks, *x* is the output of CNN-based encoder, whose contexture representations in feature maps are relatively short-range than transformer's. With Resnet, the element-wise addition of *f*(*x*) and *x* can directly fuse the short-range spatial relations from CNN-based encoder and long-range spatial relations from transformer-based bottleneck. Additionally, unlike neural network layers, element-wise addition is a math operation with no more memory cost and negligible computation time cost. The decoder part contains amounts of upsampling layers and skip connection to progressively recover semantic information as well as resolution. The first upsampling layer is implemented by interpolation while the other upsampling layers adapt the form of deconvolution with stride set to 2. At level *i* ∈ [1, 5], the encoder block *D*_*i*_ doubles the spatial resolution, followed by skip connection to fuse high-level (from *D*_*i*_) and low-level (from encoder block *E*_*i*_) contextual representation so as to segment the desired tumor regions. For a better supervision performance, the outputs of *D*_*i*_ where *i* ∈ [1, 3] are fed into 1 × 1 × 1 convolution layer and sigmoid layer to predict segmentation maps with different resolution. Accordingly, the ground truth is downsampled to different shapes such that they match the shapes of those segmentation maps.

**Table 1:** The detailed structure of proposed generator.

**Stage**	**Name**	**Details**	**Output size**
**Encoder**	E1	[Conv3, IN, LeReLU, Dropout]	64*160*192*160
		[Conv3, IN, LeReLU, Dropout]	
	E2	[Conv3(stride2), IN, LeReLU, Dropout]	96*80*96*80
		[Conv3, IN, LeReLU, Dropout]	
	E3	[Conv3(stride2), IN, LeReLU, Dropout]	128*40*48*40
		[Conv3, IN, LeReLU, Dropout]	
	E4	[Conv3(stride2), IN, LeReLU, Dropout]	192*20*24*20
		[Conv3, IN, LeReLU, Dropout]	
	E5	[Conv3(stride2), IN, LeReLU, Dropout]	256*10*12*10
		[Conv3, IN, LeReLU, Dropout]	
	E6	[Conv3(stride2), IN, LeReLU, Dropout]	384*5*6*5
		[Conv3, IN, LeReLU, Dropout]	
	E7	[Conv3(stride2), IN, LeReLU, Dropout]	512*3*3*3
		[Conv3, IN, LeReLU, Dropout]	
Transformer	ResTransBlock	Reshape	512*3*3*3
		PE	
		Transformer Layer*4	
		Reshape	
		Resnet	
Decoder	D6	Upsample	384*5*6*5
		[Conv3, IN, LeReLU, Dropout] x 2	
	D5	Deconv	256*10*12*10
		Concat	
		[Conv3, IN, LeReLU, Dropout]	
		[Conv3, IN, LeReLU, Dropout]	
	D4	Deconv	192*20*24*20
		Concat	
		[Conv3, IN, LeReLU, Dropout]	
		[Conv3, IN, LeReLU, Dropout]	
	D3	Deconv	128*40*48*40
		Concat	
		[Conv3, IN, LeReLU, Dropout]	
		[Conv3, IN, LeReLU, Dropout]	
	Output3	Conv1 + Sigmoid	4*40*48*40
	D2	Deconv	96*80*96*80
		Concat	
		[Conv3, IN, LeReLU, Dropout]	
		[Conv3, IN, LeReLU, Dropout]	
	Output2	Conv1 + Sigmoid	4*80*96*80
	D1	Deconv	64*160*192*160
		Concat	
		[Conv3, IN, LeReLU, Dropout]	
		[Conv3, IN, LeReLU, Dropout]	
	Output1	Conv1 + Sigmoid	3*160*192*160

**Figure 2:**
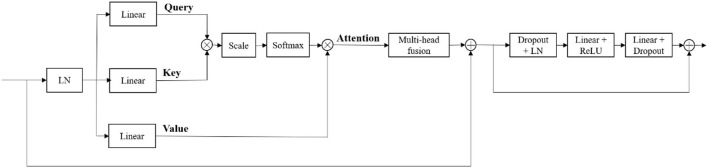
The structure of transformer layer.

Our generator's vital part is the transformer with Resnet module. As shown in Table 1, our transformer with Resnet module consists of transformer block and Resnet, while transformer block is composed of position encodings module, several transformer layers depicted in Figure 2 and features projection module. To make use of the order of the input sequence reshaped from bottom layer feature maps, we introduce a learnable positional encoding vector to represent some information about position of tokens in the sequence, instead of sine and cosine functions. After position encoding and normalization, the input sequence is fed into three different linear layers to create queries, keys, and values. Then, we compute the dot products of keys with queries. To avoid extremely small gradients after softmax function, we scale the dot-products by a factor related to dimensions of queries, as shown in Equation 5. Multiplying scaled weights with values, we obtain a single attention output, which is then concatenated with other heads' outputs to produce the multi-head attention outputs. Subsequently, normalization, dropout, and multi-layer perception (MLP) layers are utilized to produce the transformer layer's ultimate output. While convolution layers have local connections, shared weights, and translation equivariance, attention layers are global. We take advantage of both by residual connection to learn both short-range and long-range spatial relations with no more memory cost and negligible computational time cost.

### 3.3. Discriminator and loss function

To distinguish the difference between the prediction and GT, the discriminator D extracts features of GT and prediction to calculate *L*_1_ norm distance between them. The discriminator is composed of six similar blocks. Each of these blocks consists of a 3 × 3 × 3 convolution layer with a stride of 2, a batch normalization layer and a LeakyReLU activation layer. Instead of only using the final output of D, we leverage the *j*th output feature $f_{j}^{i}\left(x\right)$ extracted by *i*th (*i* ∈ [1, 2, …, *L*]) layers from image x to calculate multi-scale *L*_1_ loss ℓ_*D*_ as follows:

(6) $$\begin{array}{l} \ell_{D}\left(x,x^{{\;}^{\prime }}\right)=\frac{1}{L*M}\overset{L}{\underset{i=1}{\sum }}\overset{M}{\underset{j=1}{\sum }}||f_{j}^{i}\left(x\right)-f_{j}^{i}\left(x^{{\;}^{\prime }}\right)|{|}_{1} \end{array}$$

where M denotes the number of extracted features of a layer in D.

Referring to the loss function of GAN (Goodfellow et al., 2014), our loss function of the whole adversarial process is described as follows:

(7) $$\begin{array}{r} \begin{array}{c} \underset{\theta_{G}}{\min}\underset{\theta_{D}}{\max}L\left(\theta_{G},\theta_{D}\right)={𝔼}_{x\sim P_{data}}\left(\ell_{D}\left(G\left(x\right),y\right)\right)\\ +{𝔼}_{x\sim P_{data}}\left(\ell_{deep\text{\_}bce\text{\_}dice}\left(G\left(x\right),y\right)\right) \end{array} \end{array}$$

where *x*, *y* denote the input image and ground truth, respectively, ℓ_*deep*_*bce*_*dice*_ denotes that the segmentation maps of generator are used to calculate the BCE loss together with the Dice loss under deep supervision. Concretely, ℓ_*deep*_*bce*_*dice*_ is a weighted sum of ℓ_*deep*_*bce*_*dice*_ (*p*_*i*_, *y*_*i*_),*i* ∈ [1, 2, 3] for prediction *p*_*i*_ and mask *y*_*i*_ where *i* denotes the ith level of decoder (*D*_*i*_).

The detailed training process is presented in Algorithm 1, which interprets the procedure of sampling data and following updating discriminator and generator with corresponding loss function respectively.

**Algorithm 1:** The detailed training process. ℓ_*deep*_*bce*_*dice*_ represents BCE Dice loss with deep supervision, ℓ_*D*_ represents multi-scale *L*_1_ loss.

1: **for** number of training epoches **do**
2: **for** steps of training discriminator **do**
3: Get n input images from *p*_*data*_ {*x*^1^, …, *x*^*n*^} and corresponding labels {*y*^1^, …, *y*^*n*^}.
4: Update discriminator by maximizing the loss below: $$\begin{array}{l} \frac{1}{n}\overset{n}{\underset{i=1}{\sum }}\left[\ell_{D}\left(G\left(x^{i}\right),y^{i}\right)\right] \end{array}$$
5: Clip the weights of discriminator.
6: **end for**
7: Get n input images from *p*_*data*_ {*x*^1^, …, *x*^*n*^} and corresponding labels {*y*^1^, …, *y*^*n*^}.
8: Update generator by minimizing the loss below: $$\begin{array}{l} \frac{1}{n}\overset{n}{\underset{i=1}{\sum }}\left[\ell_{deep\text{\_}bce\text{\_}dice}\left(G\left(x^{i}\right),y^{i}\right)+\ell_{D}\left(G\left(x^{i}\right),y^{i}\right)\right] \end{array}$$
9: **end for**

## 4. Experimental results

### 4.1. Dataset

**Figure 3:**
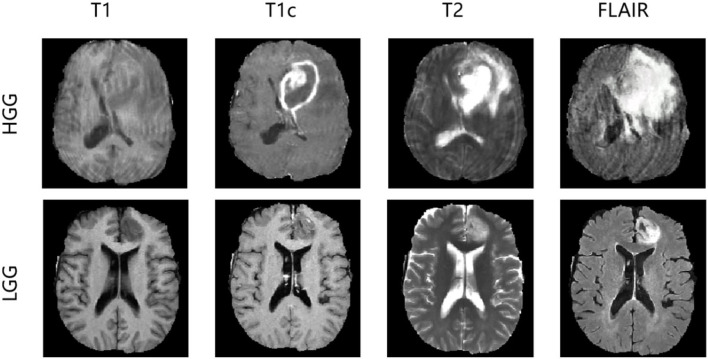
HGG and LGG cases with four modalities: T1, T1c, T2, FLAIR on BRATS2015 dataset.

In the experiments, we evaluated our method using the Brain Tumor Image Segmentation Challenge 2015 (BRATS2015) dataset. In BRATS2015, the training dataset contains manual annotation by clinical experts for 220 patient cases with high-grade glioma (HGG) and 55 patient cases with low-grade glioma (LGG), whereas 110 patient cases are supplied in the online testing dataset without annotation. Four 3D MRI modalities—T1, T1c, T2, and FLAIR—are used for all patient cases, as depicted in Figure 3. Each modality has the origin size 240 × 240 × 155 with the same voxel spacing. The ground truth has five classes: background (label 0), necrosis (label 1), edema (label 2), non-enhancing tumor (label 3), and enhancing tumor (label 4). We divided the 275 training cases into a training set and a validation set with the ratio 9:1 both in HGG and LGG. During training and validation, we padded the origin size 240 × 240 × 155 to size 240 × 240 × 160 with zeros and then randomly cropped into size 160 × 192 × 160, which makes sure that the most image content is included.

### 4.2. Evaluation metric

To evaluate the effectiveness of a segmentation method, the most basic thing is to compare it with the ground truth. In the task of brain tumor segmentation, there are three main evaluation metrics compared with the ground truth: Dice, Positive predictive Value (PPV), and Sensitivity, defined as follows:

(8) $$\begin{array}{l} Dice\left(P,T\right)=\frac{1}{2}\times \frac{\left|P_{1}⋂T_{1}\right|}{\left(\left|P_{1}|+|T_{1}\right|\right)} \end{array}$$

(9) $$\begin{array}{l} PPV\left(P,T\right)=\frac{\left|P_{1}⋂T_{1}\right|}{\left|P_{1}\right|} \end{array}$$

(10) $$\begin{array}{l} Sensitivity\left(P,T\right)=\frac{\left|P_{0}⋂T_{0}\right|}{\left|T_{0}\right|} \end{array}$$

where *P* represents the prediction segmented by our proposed methods, *T* represents the corresponding ground truth. *P*_1_ and *T*_1_ denote the brain tumor region in *P* and *T*, *P*_0_ and *T*_0_ denote the other region except brain tumor in *P* and *T*, respectively, |·| calculates the number of voxels inside region, ∩ calculates the intersection of two regions. When Dice is larger, PPV and Sensitivity are larger at the same time, the predicted segmentation is considered to be more similar to ground truth, proving that the segmentation method is more effective.

### 4.3. Implementation details

Experiments were run on NVIDIA A100-PCIE (4 × 40GB) system for 1,000 epochs (about 3 days) using the Adam optimizer (Kingma and Ba, 2014). The target segmentation maps are reorganized into three tumor subregions: whole tumor (WT), tumor core (TC), and enhancing tumor (ET). The initial learning rate is 0.0001 and batch size is 4. The data augmentation consists of three parts: (1) padding the data from 240 × 240 × 155 to 240 × 240 × 160 with zeros; (2) randomizing the data's cropping from 240 × 240 × 160 to 160 × 192 × 160; (3) random flipping the data across three axes by a probability with 0.5. Impacted by the volumetric input size, the number of parameters of our network is larger than common 2D networks, generator: 58.0127M, transformer blocks inside generator: 11.3977M, discriminator: 75.4524M. Both the Dice loss in deep supervision and multi-scale *L*_1_ loss are employed to train the network in competing progress. In inference, we converted the transformed three subregions (WT, TC, ET) back to the original labels. Specially, we replace the enhancing tumor with necrosis when the possibility of enhancing tumor in segmentation map is less than the threshold, which is chosen according to the online testing scores.

### 4.4. Impact of the number of generators and discriminators

As the BRATS2015 is a multi-label segmentation task, our architecture can be implemented with schemes where the number of generators and discriminators are different. Each implementation scheme in Table 2 is specifically described as follows:

- 1G-1D. The network is composed of one generator and one discriminator. The generator outputs three-channel segmentation maps corresponding to three brain tumor subregions, while the discriminator is fed with three-class masked images concatenated in channel dimension.
- 1G-3D. The network is composed of one generator and three discriminators. The generator outputs three-channel segmentation maps while the discriminators output three one-channel maps, each for one class.
- 3G-3D. The network is composed of three generators and three discriminators. Each generator or discriminator is built for one class. There are three pairs of generators and discriminators, indicating that each pair is trained independently for one class.

**Table 2:** Results of different number of generators and discriminators.

**Method**	**Dice**			**Positive predictive value**			**Sensitivity**		
	**Whole**	**Core**	**Enha**.	**Whole**	**Core**	**Enha**.	**Whole**	**Core**	**Enha**.
1G-3D	**0.85**	**0.73**	**0.63**	**0.83**	**0.79**	0.59	**0.90**	**0.73**	**0.73**
1G-1D	0.84	0.72	0.62	0.82	0.78	0.58	0.89	0.72	0.71
3G-3D	0.81	0.68	0.60	0.83	0.74	**0.62**	0.84	0.70	0.63

Whole, whole tumor; Core, tumor core; Enha., enhancing tumor.

### 4.5. Evaluating the transformer with Resnet module

To evaluate the effectiveness of the transformer with Resnet module, we conduct some ablation experiments. We design the bottom layer of our proposed generator with different schemes as follows:

- Transformer with Resnet. The bottom layer is composed of Transformer with Resnet we proposed.
- Transformer w/o Resnet. The bottom layer is composed of Transformer block, ranging from projection, position embedding to transformer layers, without shortcut crossing them.
- CNN with Resnet. The bottom layer is composed of convolutional layers together with a shortcut crossing them.
- Shortcut. The bottom layer is simply a shortcut connection from the encoder part to the decoder part.

The comparation results are shown in Table 3. From the results, we demonstrate the transformer's superiority and irreplaceability, and we can conclude that transformer with Resnet module make the best of features from transformer block and convolutional encoder to improve the segmentation performance.

**Table 3:** Results of different bottom layer in generator.

**Method**	**Dice**			**Positive predictive value**			**Sensitivity**		
	**Whole**	**Core**	**Enha**.	**Whole**	**Core**	**Enha**.	**Whole**	**Core**	**Enha**.
Transformer with Resnet	**0.85**	**0.73**	**0.63**	**0.83**	**0.79**	0.59	0.90	**0.73**	**0.73**
Transformer w/o Resnet	0.85	0.71	0.61	0.83	0.79	0.60	0.90	0.69	0.68
CNN with Resnet	0.83	0.68	0.58	0.80	0.78	0.58	**0.91**	0.66	0.62
Shortcut	0.82	0.67	0.60	0.82	0.77	**0.63**	0.87	0.67	0.63

Whole, whole tumor; Core, tumor core; Enha., enhancing tumor.

### 4.6. Evaluating the CNN-based discriminator

We select the CNN-based discriminator instead of the transformer-based one as our final discriminator in our architecture, due to our opinion that transformer-based multi-layers discriminator requires huge datasets to support pre-training. To prove that, we conduct ablation experiments to compare their performance by training from scratch. The transformer-based discriminator is implemented using the inspiration of Jiang et al. (2021). Table 4 shows the results on BRATS2015 testing dataset using different discriminators, from which our CNN-based discriminator shows its superior capability of classifying the ground truth and segmentation outputs from scratch. Without pre-training, the CNN-based discriminator appears to be better than the transformer-based one.

**Table 4:** Results of different discriminators training from scratch.

**Method**	**Dice**			**Positive predictive value**			**Sensitivity**		
	**Whole**	**Core**	**Enha**.	**Whole**	**Core**	**Enha**.	**Whole**	**Core**	**Enha**.
CNN-based	**0.85**	**0.73**	**0.63**	**0.83**	**0.79**	**0.59**	**0.90**	**0.73**	**0.73**
Transformer-based	0.79	0.66	0.58	0.79	0.77	0.55	0.86	0.64	0.66

Whole, whole tumor; Core, tumor core; Enha., enhancing tumor.

### 4.7. Evaluating the loss function

In this section, we evaluate the effectiveness of the loss function in our proposed methods. As shown in Equation 7, our loss function is divided into two parts: the deep supervision loss and multi-scale *L*_1_ loss. We conduct two ablation experiments: one model with single-scale *L*_1_ loss, the other model without deep supervision loss. It is worth noting that the implementation of these models is the same as 1G-3D where the network consists of one generator and three discriminators and employs the transformer with Resnet module in the bottom layer. From Table 5, we find that our loss function achieves better performance under the same other experimental environment.

**Table 5:** Results of different loss function.

**Method**	**Dice**			**Positive predictive value**			**Sensitivity**		
	**Whole**	**Core**	**Enha**.	**Whole**	**Core**	**Enha**.	**Whole**	**Core**	**Enha**.
Our method	**0.85**	**0.73**	**0.63**	**0.83**	**0.79**	**0.59**	**0.90**	**0.73**	**0.73**
w/o deep supervision	0.85	0.72	0.61	0.83	0.78	0.57	0.90	0.73	0.71
Single-scale *L*_1_ loss	0.84	0.72	0.61	0.82	0.78	0.58	0.89	0.72	0.71

Whole, whole tumor; Core, tumor core; Enha., enhancing tumor.

**Figure 4:**
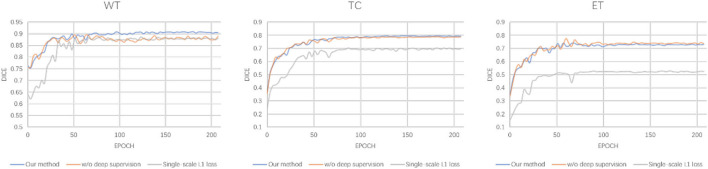
Detailed evaluation curves of different loss function.

The detailed segmentation evaluation scores curves with different loss function are depicted in Figure 4. It is clear that the segmentation performance of all approaches steadily increases as the number of epochs increases until it reaches a steady state. Ranging from WT, TC to ET, our method shows an increasing performance boost over other methods. As a consequence, our method yields the best results in all evaluation metrics listed in Table 5.

### 4.8. Comparison with other methods

To obtain a more robust prediction, we ensemble 10 models trained with the whole training dataset to average the segmentation probability maps. We upload the results of our methods on the BRATS2015 dataset and get the testing scores computed *via* the online evaluation platform, as listed in Table 6.

**Table 6:** Performance of some methods on BRATS2015 testing dataset.

**Method**	**Dice**			**Positive predictive value**			**Sensitivity**		
	**Whole**	**Core**	**Enha**.	**Whole**	**Core**	**Enha**.	**Whole**	**Core**	**Enha**.
UNET (Ronneberger et al., 2015)	0.80	0.63	0.64	0.83	0.81	**0.78**	0.80	0.58	0.60
ToStaGAN (Ding et al., 2021)	**0.85**	0.71	0.62	0.87	**0.86**	0.63	0.87	0.68	0.69
3D Fusing (Zhao et al., 2018)	0.84	**0.73**	0.62	0.89	0.76	0.63	0.82	**0.76**	0.67
FSENet (Chen et al., 2018)	**0.85**	0.72	0.61	0.86	0.83	0.66	0.85	0.68	0.63
SegAN (Xue et al., 2018)	**0.85**	0.70	**0.66**	**0.92**	0.80	0.69	0.80	0.65	0.62
Our method	**0.85**	**0.73**	0.63	0.83	0.79	0.59	**0.90**	0.73	**0.73**

Whole, whole tumor; Core, tumor core; Enha., enhancing tumor.

**Figure 5:**
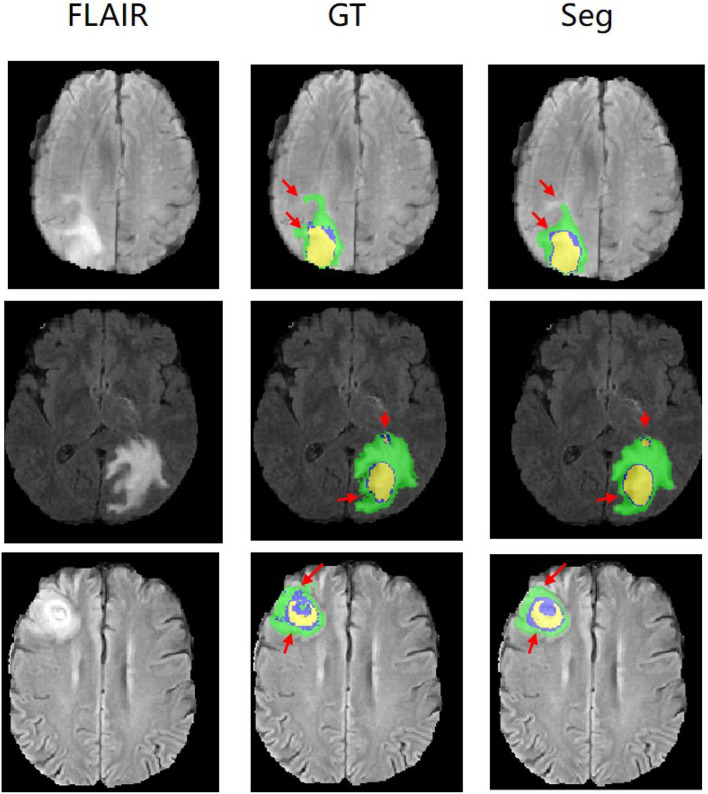
Experimental results with corresponding slices on BRATS2015 validation set. The red arrows locate the mainly different regions between GT and segmentation results.

Figure 5 shows our qualitative segmentation output on BRATS2015 validation set. This figure illustrates different slices of different patient cases in ground truth and predictions separately.

### 4.9. Qualitative analysis

**Figure 6:**
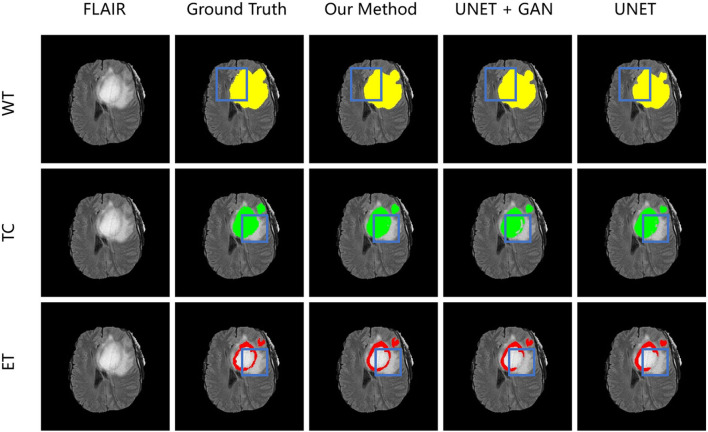
From left to right is the visualization results of an original image in FLAIR modality, ground truth, model in our method, model in the form of UNET + GAN, model UNET. From up to down in each column is three segmentation maps predicted with the same method. The blue boxes outline the difference between results from different methods.

To demonstrate the performance of our proposed method, we randomly choose a slice of one patient on BRATS2015 validation set to visualize and compare the result in Figure 6. In Figure 6, images in the same column are produced from the same method, and images in the same row are belonging to the same segmentation label. Concretely, the column FLAIR represents the original image with modality of FLAIR, while other columns are segmentation maps with corresponding categories and colors: WT is yellow, TC is green, and ET is red. The column UNET represents that the corresponding three segmentation maps are inferenced with model UNET. The model of the column UNET plus GAN is built based on UNET, with an addition of GAN, where the generator is UNET with deep supervision and discriminator is a CNN-based network with multi-scale *L*_1_ loss. A deep insight of Figure 6 reveals that with the help of deep supervision and multi-scale *L*_1_ loss, the UNET+GAN method segments fuller edges and richer details than UNET method. When the transformer block is applied, our method produces more smooth borders on the tumor core regions, and more complete contours on enhancing tumor regions. The reason for this improvement seems to be that the transformer with Resnet module can effectively model the short-range and long-range dependency, and collect both local and global contexture representation information. Owing to more complete features, our method achieves the better performance.

### 4.10. Generalization on other datasets

To evaluate generalization of our proposed method, we conduct additional experiments on other datasets relative to brain tumor segmentation, BRATS2018 and BRATS2020, which are composed of more practical patient cases. These datasets differ from BRATS2015 dataset in labels, number of cases and difficulty. The detailed inference performance are listed in Tables 7, 8. On BRATS2018 validation dataset, our proposed method achieves Dice score of 0.7686, 0.9021, and 0.8089, and Hausdorff (HD) of 5.7116, 5.4183, and 9.4049 mm on ET, WT, and TC, respectively. On BRATS2020 validation dataset, our method also realizes Dice score of 0.708, 0.903, and 0.815 and HD of 37.579, 4.909, and 7.494 mm on ET, WT, and TC, respectively. These excellent scores reveal the great generalization of our transformer-based generative adversarial network.

**Table 7:** Comparison to other methods on BRATS2018 validation dataset.

**Method**	**Dice(mean)**			**Hausdorff(mm)**		
	**Enha**.	**Whole**	**Core**	**Enha**.	**Whole**	**Core**
Myronenko (2018)	0.7664	0.8836	**0.8154**	3.7731	5.9044	**4.8091**
Hu et al. (2019)	0.7178	0.8824	0.7481	**2.8000**	**4.4800**	7.0700
Chandra et al. (2018)	0.7406	0.8719	0.7990	5.5757	5.0379	9.5884
Liu (2018)	0.7639	0.8958	0.7905	4.0714	4.4924	8.1971
Our method	**0.7686**	**0.9021**	0.8089	5.7116	5.4183	9.4049

Whole, whole tumor; Core, tumor core; Enha., enhancing tumor.

**Table 8:** Comparison to other methods on BRATS2020 validation dataset.

**Method**	**Dice(mean)**			**Hausdorff(mm)**		
	**Enha**.	**Whole**	**Core**	**Enha**.	**Whole**	**Core**
Tang et al. (2020)	0.703	0.893	0.790	**34.306**	**4.629**	10.071
Zhou et al. (2022)	0.647	0.818	0.759	44.400	10.000	14.600
Anand et al. (2020)	**0.710**	0.880	0.740	38.310	6.880	32.000
Zhang et al. (2021)	0.700	0.880	0.740	38.600	7.000	30.200
Our method	0.708	**0.903**	**0.815**	37.579	4.909	**7.494**

Whole, whole tumor; Core, tumor core; Enha., enhancing tumor.

## 5. Discussion and conclusion

In this paper, we explored the application of a transformer-based generative adversarial network for segmenting 3D MRI brain tumors. Unlike many other encoder–decoder architectures, our generator employs a transformer with Resnet module to effectively model the long-distance dependency in a global space, simultaneously inheriting the advantage of CNNs for learning the capability of local contexture representations. Moreover, the application of deep supervision improves the flowability of gradient to some extent. Our discriminator is applied to measure the norm distance of hierarchical features from predictions and masks. Particularly, we calculate multi-scale *L*_1_ loss between the generator segmentation maps and ground truth. Experimental results on BRATS2015, BRATS2018, and BRATS2020 datasets show a better performance of our proposed method in comparison of other state-of-the-art methods, which proves the superior generalization of our method in brain tumor segmentation.

## Data availability statement

The dataset BRATS2015 (Menze et al., 2014; Kistler et al., 2013) for this study can be found in the https://www.smir.ch/BRATS/Start2015. The dataset BRATS2018, BRATS2020 (Menze et al., 2014; Bakas et al., 2017, 2018) and online evaluation platform can be found in this https://ipp.cbica.upenn.edu.

## Author contributions

LH: conceptualization, methodology, software, project administration, writing—original draft, writing—review, and editing. EZ: medical expert. LC: validation and project administration. ZW and BZ: supervision. SC: supervision, resources, formal analysis, and funding acquisition. All authors contributed to the article and approved the submitted version.

## Funding

This work was funded in part by the National Natural Science Foundation of China (Grant Nos. 82170374 and 82202139), and also supported in part by the Capital Medical Funds for Health Improvement and Research (CHF2020-1-1053).

## Conflict of interest

The authors declare that the research was conducted in the absence of any commercial or financial relationships that could be construed as a potential conflict of interest.

## Publisher's note

All claims expressed in this article are solely those of the authors and do not necessarily represent those of their affiliated organizations, or those of the publisher, the editors and the reviewers. Any product that may be evaluated in this article, or claim that may be made by its manufacturer, is not guaranteed or endorsed by the publisher.
